# Technological nursing interventions on nutritional status of middle-aged and older adults undergoing hemodialysis: A systematic review

**DOI:** 10.1016/j.ijnss.2025.08.008

**Published:** 2025-08-14

**Authors:** Amélia Pernas, Sara Pires, Idalina Gomes, César Fonseca, Ana Ramos

**Affiliations:** a Nefroestremoz, Estremoz, Portugal; b Nursing School of Lisbon (ESEL), Lisbon, Portugal; c Nursing Research, Innovation and Development Centre of Lisbon (CIDNUR), Lisbon, Portugal; d University of Évora, Évora, Portugal; e Comprehensive Health Research Centre (CHRC), Évora, Portugal

**Keywords:** Hemodialysis, Nutritional status, Nursing, Patients, Self-care, Technological innovation

## Abstract

**Objective:**

Malnutrition is common in hemodialysis patients, increasing mortality and significantly impacting quality of life. This study aimed to identify technological nursing interventions that promote self-care and improve the nutritional status of middle-aged and older adults undergoing hemodialysis.

**Methods:**

A systematic literature review was conducted in accordance with the Joanna Briggs Institute (JBI) and PRISMA guidelines. Searches were conducted in Medline, CINAHL, the Cochrane Library, Scopus, Web of Science, and grey literature. Studies published between 2018 and 2024, involving patients aged 40 years or more undergoing regular hemodialysis, and available in Portuguese, English, or Spanish, were included. JBI’s critical appraisal tools were used to conduct a rigorous analysis and methodological quality assessment of the articles.

**Results:**

Out of a total of 738 articles, 10 were included for analysis. Five key dimensions of technology-driven self-care interventions were established. 1) Mobile applications and digital platforms with features like nutritional databases, food logging, and personalized feedback; 2) E-learning and virtual education using social media and chat-based communication; 3) Telenursing employing a hybrid follow-up model of face-to-face, telephone, and SMS contact; 4) Educational strategies focused on nutritional status, utilizing methods such as teach-back and pictorial learning within a multidisciplinary team; and 5) Comprehensive assessment tools evaluating treatment adherence (hemodialysis, medication, diet, fluid) and laboratory markers. Significant improvements were reported across several outcomes: eight studies showed enhanced biochemical markers (e.g., phosphorus, sodium, potassium, calcium, iron, albumin, urea, and hemoglobin) and nutritional status, three demonstrated increased self-efficacy, and two reported improved quality of life.

**Conclusion:**

Integrating technology and face-to-face education enhances nutritional status, highlighting the importance of comprehensive strategies to improve treatment adherence and prevent malnutrition in hemodialysis patients.

## What is known?

• Malnutrition is highly prevalent among patients undergoing hemodialysis and is associated with increased mortality, reduced quality of life, and functional decline.
• Self-care and nutritional adherence are essential for better outcomes in hemodialysis. Still, patients—particularly older adults—often face significant barriers, including limited health literacy, comorbidities, and social determinants of health.
• Nurse-led interventions have been shown to improve self-care behaviors and treatment outcomes; however, their application to nutritional care, especially through integrating digital and technological tools, remains insufficiently explored in current literature.

## What is new?

• The review demonstrates that combining technologies like mHealth and telenursing with traditional, face-to-face education models improves clinical outcomes and dietary adherence in hemodialysis patients.
• Nurses are highlighted as key facilitators of digital health education and contribute to improving biochemical markers (e.g., phosphorus, albumin, urea), better dietary adherence, increased patient empowerment, and enhanced quality of life.
• The review reveals gaps in long-term follow-up and equity in access to digital resources, calling for future research and policy efforts to address disparities in technological adoption in renal care.

## 1 Introduction

Chronic kidney disease (CKD) affects thousands of people worldwide. It is considered a public health problem [1], associated with an increased risk of cardiovascular morbidity, premature mortality, and decreased quality of life [2]. It is estimated that by 2040, CKD will be the fifth leading cause of death and years of life lost [3]. The prevalence of CKD is notably higher in middle-aged and older adults, standing at 6 % for individuals aged 18–44 years, rising to 12 % for those aged 45–64 years, and surging to 34 % for those aged 65 years and older [4].

CKD is characterized by structural and functional loss of the kidney, lasting 3 months or more [5]. It is classified into 5 stages, from 1 to 5, associated with the degree of severity, where grade 5 corresponds to end-stage CKD with a very significant reduction in glomerular filtration rate (GFR) [6], requiring Renal Function Replacement Treatment (RRT) including hemodialysis, peritoneal dialysis, conservative treatment, and transplantation [7,8].

The population receiving dialysis has seen a significant demographic shift, with a notable increase in the number of older individuals initiating renal replacement therapy, particularly those aged 75 years and older [9]. Studies indicate a global rise in the incidence of CKD associated with the aging of the population and the growth of chronic diseases such as diabetes mellitus and hypertension, which are risk factors for CKD [[10], [11], [12]]. This global trend is driven by several interconnected factors, including an increase in overall life expectancy, more liberal acceptance criteria for older patients into dialysis programs, and reduced access to kidney transplantation as a viable treatment option for this demographic [13,14]. Hemodialysis replaces the kidney, which is essential for maintaining life and the body’s balance, as it filters the blood artificially, regularly, on fixed days, three times a week. It has a significant physical, psychological, and social impact on a person’s life [12]. Hemodialysis sessions impose severe restrictions on a person’s daily life, limiting their independence and flexibility, from session times to food and water restrictions [15]. Since the start of substitution treatment, its impact cannot be overlooked [16], the dietary pattern changes, with the need for restrictions on potassium, phosphorus, sodium, and liquids, adequate consumption of proteins, derived from quality products, avoiding processed and industrialized foods, requiring a conscious selection of foods and liquids to include in the diet [17]. Limited nutritional literacy and non-adherence to diet are also prevalent in this population [18]. These conditions can cause and be strongly associated with an impaired nutritional status, with protein-energy wasting (PEW) [19]. For all these factors, maintaining an adequate diet can be a complex challenge for the middle-aged and older adults undergoing hemodialysis treatment [20] in which nutritional status is influenced by the evolution of the chronic disease, but also by the treatment itself, which can cause loss of appetite, nausea and vomiting and changes in taste, which can be debilitating and have a profound impact on interest in food, making it challenging to consume adequate nutrients [15].

The complexity of the treatment, combined with the underlying health conditions and the inability to adhere to dietary guidelines, has a significant impact on health and quality of life [21]. High rates of altered nutritional status have been identified worldwide, with an estimated 28 to 54 percent of patients undergoing dialysis treatment affected by protein-energy malnutrition [22]. There is a direct relationship between nutritional status, basic life activities, and cognitive function, where low weight predicts deterioration in functional capacity, especially in older adults [23]. Furthermore, sarcopenia, defined by the significant loss of skeletal muscle mass and strength, affects a considerable proportion of middle-aged and older adults, up to one-third, and is a major factor contributing to their overall state of malnutrition compared to the younger patient [24,25]. Nutritional status can change rapidly, and frequent monitoring and assessing the person on hemodialysis is essential [26]. Additionally, existing nutrition guidelines often fall short, particularly for older adults, further complicating dietary protein intake and exacerbating the risk of adverse health outcomes [27].

The nutritional guidelines for hemodialysis patients, recommended by the European Society for Clinical Nutrition and Metabolism (ESPEN) Guidelines and the Kidney Disease Outcomes Quality Initiative (KDOQI) [28] developed by the National Kidney Foundation (NKF), are guiding instruments for nursing intervention, providing a nutritional care plan and individualized dietary advice, facilitating guidance on the ideal selection of food options [29]. However, caring for the person in middle-aged or older adults on hemodialysis is particularly challenging, as food choices are driven by many factors, such as comorbidities, nutritional status, socioeconomic factors, background, available budget, individual and cultural conditions, and food preferences [[30], [31], [32]]. These challenges directly affect nutritional status, making it essential to optimize it to reduce clinical complications, hospitalization rates, and mortality, with significant associated health costs [33]. In hemodialysis patients, self-care is essential for managing their health condition and adhering to treatment, which, due to its complexity, requires specialized and individualized nursing care and interventions to promote an adequate nutritional state with positive results for their health [23,28,30].

Despite advances in dialysis care, older adults undergoing this treatment often experience higher rates of mortality and hospitalization when compared to younger patient cohorts [14]. Care for middle-aged and elderly hemodialysis patients requires a more holistic, geriatric-focused approach, because health literacy is known to increase with decreasing age, which further supports the need for age-sensitive technological interventions [34]. These findings underscore the profound physiological challenges, and complex care needs inherent to providing dialysis in an aging population. Nursing interventions using technology can promote self-care in the nutritional status of middle-aged and older adults undergoing regular hemodialysis treatment, and it is essential to make sensitive nurses aware of the potential of technological innovation in the prevention and treatment of changes in nutritional status [35].

Although there is a growing concern with structuring nurse-led interventions to improve health education for hemodialysis patients [36], there is still insufficient clarity on how these interventions can be optimized by incorporating technology, especially among older populations. This systematic review aimed to identify nursing interventions that promote self-care capacity in the nutritional status of people on hemodialysis using innovation and technology.

## 2 Methods

This systematic review was conducted following the PRISMA guidelines [37]. Following the guidelines of the Joanna Briggs Institute (JBI) [38], a research question was formulated based on the PICO mnemonic [39], which drove the start of the research. Which nursing interventions use innovation and technology to promote nutritional status in middle-aged and older adults undergoing hemodialysis? The protocol for this review was registered with the International Prospective Registry of Systematic Reviews (PROSPERO) of the National Institute for Health Research in July 2024, with the registration number: CRD42024573101.

### 2.1 Search strategy

Between June and December 2024, research was conducted in the following electronic databases: Medline, CINAHL, and Cochrane Central Register of Controlled Trials via EBSCOhost, Scopus, and Web of Science. The grey literatures were searched by Google Scholar. A comprehensive search strategy used keywords and phrases related to hemodialysis, nutritional status, self-care practices, and technological nursing interventions. The search included a combination of controlled vocabulary, MeSH terms, and free-text terms to ensure a complete search. Boolean operators (AND, OR) effectively combined search terms. The detailed search strategy is set out in Appendix A.

### 2.2 Eligibility criteria

The study eligibility criteria were defined based on the PICOS framework. The review included peer-reviewed primary research (randomized controlled trials, quasi-experimental, and observational studies) and relevant grey literature evaluating a technological or digital health intervention designed to promote self-care in middle-aged and older adults (defined as ≥40 years) with CKD undergoing a regular hemodialysis program. This review is based on middle age, describing the life stage between youth and old age. We adopted a broader range, of 40–64 years, given the recent rise in technological innovations in healthcare. This period is also commonly referred to as “midlife” or “middle adulthood.” It’s important to note that the exact boundaries of middle age aren’t rigidly defined, either in common language or academic literature [40].

Studies were required to report on at least one of the following outcomes: biochemical markers, nutritional status, treatment adherence, self-efficacy, or quality of life. The search was limited to articles published in English, Portuguese, or Spanish between January 2018 and 2024. This timeframe was chosen to focus on the most current digital health interventions. Given the sector’s rapid technological evolution, studies published before 2018 were excluded, as their findings may not accurately reflect the contemporary landscape of digital tools. Studies were excluded if they are review articles, editorials, case reports, or focused on pediatric populations or non-technological interventions.

Exclusion with a population that included children or young adults, as a protocol methodology, opinion articles, or secondary studies, as well as those without full text. Studies that did not address patients undergoing hemodialysis treatment and did not focus on the nutritional status of this population were excluded.

### 2.3 Evaluation of risk of bias and methodological quality of studies

To minimize the risk of bias, two researchers (A. Pernas & A. Ramos) initially independently assessed the full text of eligible articles, which were subsequently included in the review. JBI’s critical appraisal tools were used to conduct a rigorous analysis and methodological quality assessment of the articles. According to Barker et al. [41], robust systematic reviews provide a rigorous and valid synthesis of the best available evidence on the effects of interventions or treatments. For randomized controlled trials, we employed the 13-item JBI tool to evaluate key areas such as the randomization process, allocation concealment, baseline group comparability, blinding of participants and personnel, consistency in outcome measurement, completeness of follow-up, and the appropriateness of statistical analysis [42]. Quasi-experimental studies were appraised using the 9-item JBI checklist, focusing on the clarity of the cause-and-effect relationship, the comparability of participant groups, the management of confounding factors, the reliability of outcome measurements, and the completeness of follow-up [43]. Finally, the quality of analytical cross-sectional studies was assessed with the 8-item JBI checklist. This evaluation centered on the clarity of sample inclusion criteria, the detailed description of subjects and settings, the validity and reliability of exposure and outcome measurements, and the identification and management of confounding variables [44]. Each item was rated as “yes,” “no,” “unclear,” or “not applicable.” For scoring purposes, “yes” responses were assigned a value of 1, while “no”, “unclear” and “not applicable” were scored as 0. Final scores were converted into percentages to facilitate comparison across studies. A higher percentage indicated that more methodological criteria were met, reflecting higher overall methodological quality.

Two researchers conducted this evaluation independently (A. Pernas & A. Ramos). Articles were classified based on quality, according to scores calculated as the percentage of “yes” responses, where scores above 80 % were considered high quality, 60 %–80 % moderate quality, and below 60 % low quality [45].

### 2.4 Data extraction and synthesis

The final studies were selected for inclusion in the systematic literature review according to the inclusion and exclusion criteria. The Rayyan© platform automatically eliminated duplicates. To identify relevant studies for this review and those that answered the proposed objective, an initial screening by title and abstract was carried out independently by two researchers (A. Pernas & A. Ramos). Subsequently, the two researchers subjected these pre-selected articles to a full-text reading and detailed analysis, with a concordance rate of over 80 percent. In cases of disagreement, the opinion of a third researcher (I. Gomes) was sought. For data extraction from the selected articles, tables were used containing the following information: authors, year of publication, country, objective, study design, populations, duration of interventions, type of intervention, scales used, results, and main conclusions. The authors developed this instrument. Subsequently, a narrative synthesis was developed, incorporating all significant findings that emerged from the full-text analysis of each article. A refined figure was constructed in the final stage to summarize the data.

## 3 Results

### 3.1 Study selection

**Fig. 1:**
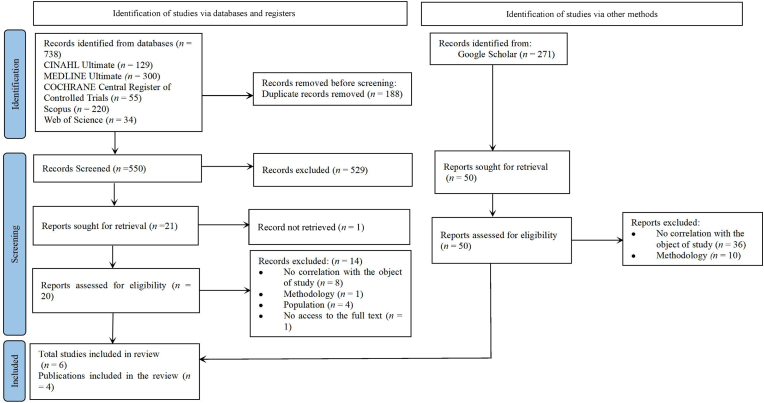
PRISMA flowchart of the article selection process.

A total of 981 articles were identified, with 738 originating from electronic database searches. The search identified and removed 188 duplicate articles from scientific databases. After this step, the titles and abstracts of 550 articles were reviewed, of which 529 were excluded for not addressing the formulated research question or failing to meet the review’s eligibility criteria. Seventeen articles remained and were thoroughly analyzed with full-text retrieval. Of these, one article could not be accessed in full text, and nine were excluded: five due to a lack of correlation with the study’s objective, one due to methodological issues, and four because the population was not eligible. As a result, seven articles from electronic databases and four from Google Scholar were included, as shown in Fig. 1.

### 3.2 Study characteristics

The 10 selected studies [[46], [47], [48], [49], [50], [51], [52], [53], [54], [55]], aligned with the review’s objective, were conducted in various countries, namely Australia (*n* = 1), Saudi Arabia (*n* = 1), Iran (*n* = 4), China (*n* = 3), and South Korea (*n* = 1). The sample analyzed included 988 patients undergoing hemodialysis. Most studies involved patients with a mean age within the middle-aged range [[49], [50], [51], [52],^55^], while Mozafari [47] focused exclusively on older adults. Chiang et al, [54] presented the most diverse age range. Although the average age of participants in the study by Arad et al. [48] and Hosseini et al. [53] was slightly below 40 years, it was decided to include it in the review, considering that it is very close to this threshold. This decision is supported by scientific evidence on the use of technology in this population is still emerging.

The review includes six articles incorporating interventions using innovation and technology [46,48,49,[53], [54], [55]], while three articles focus on interventions without technological resources [47,51,52]. Additionally, one article describes the key predictive factors of malnutrition in this population, complementing the topic discussed [50]. It is essential to note that only high- and moderate-quality studies were included in this review (Table 1). The risk of bias for all included articles was assessed using the JBI Checklist, demonstrating high or medium quality (ranging from 75.0 % to 92.3 %), with an average score of 85.3 %. A general analysis of this assessment is presented in the table below, while detailed results can be found in Appendix B.

**Table 1:** Results of the methodological quality assessment of the included manuscripts.

Study	Total items	Score (%)	Level
JBI Critical Appraisal Tool for randomized controlled trials			
Naseri-Salahshour et al. (2020) [46]	12/13	92.3	High
Mozafari et al. (2024) [47]	11/13	84.6	High
Arad et al. (2021) [48]	12/13	92.3	High
Pack & Lee (2021) [49]	10/13	76.9	Medium
JBI Critical appraisal tool of analytical cross-sectional studies			
Azzeh et al. (2022) [50]	7/8	87.5	High
JBI Critical appraisal tool of quasi-experimental studies			
Mackay et al. (2019) [51]	6/8	75.0	Medium
Tang & Fu (2021) [52]	8/9	88.9	High
Hosseini et al. (2023) [53]	5/6	83.3	High
Chiang et al. (2021) [54]	8/9	88.8	High
Wang & Hu (2024) [55]	8/9	88.9	High

### 3.3 Mobile applications and digital platforms

Hosseini et al. [53] conducted a quasi-experimental study demonstrating that an educational application showed significant potential in increasing self-efficacy and self-care by up to 50 %. This contributed to better stress management and the development of positive attitudes among hemodialysis patients, with effects observed over six months. Chiang et al. [54] implemented an individualized phosphorus control program via a smartphone application, achieving better control of serum levels. This intervention resulted in reductions in potassium levels (up to 0.5 mEq/L) and phosphorus levels (up to 0.97 mg/dL), as well as an increase in dietary knowledge among patients.

Internet-based cognitive behavioral therapy (ICBT) via WeChat in hemodialysis patients has improved nutritional indicators, such as albumin and pre-albumin levels, which are key markers of nutritional status [55]. They typically feature educational videos, behavioral training tools (e.g., food intake tracking), relaxation techniques, and psychological counseling sessions, all delivered through a digital interface. Individualized guidance and real-time monitoring are often key components. Comprehensive evaluation includes a wide range of clinical indicators (electrolytes, nutritional markers, waste products), nutritional risk and inflammation scores, and psychological assessments (appetite, KAP questionnaire) measured before and after the intervention.

Evidence strongly supports the efficacy of these apps. Pack & Lee [49] conducted a randomized controlled trial (RCT) demonstrating that a smartphone-based dietary self-management program, featuring automated feedback and personalized interactions, significantly improved phosphorus and potassium levels, enhanced self-efficacy, and increased the quality of life of hemodialysis patients. This highlights the app’s potential to empower patients with independent dietary control and immediate, actionable feedback.

The apps are meticulously designed with features like local nutritional databases (for checking phosphate-rich foods), simplified portion size evaluation, “health scores” based on dietary ratios, recipes, food preparation strategies, and food log analysis with individualized adjustments. Nurses often provide initial in-person training on app usage and continuous online support. The interventions are typically structured in phases, from introduction and practice to long-term maintenance. Success of the intervention is measured through changes in specific biochemical parameters (e.g., phosphorus, potassium), self-efficacy scales, and quality of life questionnaires (e.g., KDQOL-SF) at various time points (e.g., 8 and 12 weeks).

### 3.4 E-learning and virtual education

Naseri-Salahshour et al. [46] focused on the effects of a virtual nutritional education program through a Telegram channel for four weeks, impacting the reduction of sodium, phosphorus, potassium, and magnesium levels, and improving quality of life. The intervention focuses on dietary considerations specific to hemodialysis patients (e.g., fluid restrictions, protein, mineral control). This content is then disseminated regularly through accessible digital channels. For instance, Naseri-Salahshour et al. [46] sent messages twice weekly over four weeks, incorporating patient feedback and discussions. Effectiveness is assessed by comparing pre- and post-intervention scores on quality-of-life questionnaires and analyzing changes in serum electrolyte levels.

### 3.5 Telenursing

Arad et al. [48] conducted an RCT in which the intervention included a nurse-led telephone and SMS follow-up educational program combining elements of face-to-face education and distance support through basic technologies. This program intensified adherence to treatment in all dimensions assessed: a) compliance with the number of hemodialysis sessions, b) adherence to the medication regime, fluid restriction, and c) dietary recommendations. Changes for the better were also seen in laboratory indicators, with a reduction in sodium, potassium, creatinine, phosphorus, and urea and an increase in calcium, iron, albumin, and hemoglobin. It proved to be an accessible and replicable strategy, with reduced complications and improved quality of life.

### 3.6 Educational strategies targeting nutritional status

Tang & Fu [52] investigated the effectiveness of the Transtheoretical Model combined with Nutritional Intervention (TTMNI) as part of routine nursing care without using advanced digital devices. The study observed improved nutritional indices and adherence to dietary and nutritional recommendations. Mozafari et al. [47] compared two in-person educational methods (teach-back vs. pictorial image-based learning) in older adults with low health literacy. Both methods enhanced nutritional knowledge, but the pictorial image approach proved more effective. Mackay et al. [51] described implementing an evidence-based dietary care model developed using the Knowledge-to-Action Framework. This approach emphasized in-person training and multidisciplinary team involvement to monitor and promote the nutritional status of hemodialysis patients. Results showed a reduction in malnutrition prevalence, decreasing from 28 % at baseline to 23 % after six months and 20 % after 12 months. Although the difference was not statistically significant, the positive trend suggests that specialized teams and nutritional counseling services can improve patients’ nutritional status. Azzeh et al. [50] conducted an observational study on hemodialysis of middle-aged and older adults in two dialysis centers, with a sample of 211 patients. The study identified several key predictors of malnutrition in this population:

• Prolonged time on hemodialysis (more than 4 years): This was the most significant factor, with patients undergoing hemodialysis for extended periods having a substantially higher risk of malnutrition.
• Polypharmacy (use of more than four medications): Patients taking a high number of medications for chronic conditions were almost three times more likely to experience malnutrition, indicating a strong and consistent association.
• Low muscle strength and reduced handgrip strength: Physical indicators, such as decreased muscle strength and lower handgrip strength, were significantly associated with malnutrition, reflecting muscle mass loss and physical frailty in hemodialysis patients.
• Unemployment: This socioeconomic factor was also identified as a predictor of malnutrition, with unemployed patients showing an increased probability of malnutrition. However, this association’s strength was lower than that of the other factors.

Although this study does not directly reference nursing interventions, it provides valuable contributions by serving as a foundation for individualizing nursing interventions, contextualizing the clinical environment in which these interventions are applied, and guiding nurses in risk stratification for malnutrition. This stratification allows interventions—whether technological, traditional, or combined—to be tailored to the specific needs of patients, thereby enhancing the effectiveness of self-care in maintaining nutritional status. A summary of the characteristics of the included studies and the main findings is presented in Appendix C.

### 3.7 Nutritional status assessment tools

Four studies employed validated nutritional assessment tools in middle-aged or older adults undergoing hemodialysis. Mackay et al. [51] utilized the Patient-Generated Subjective Global Assessment (PG-SGA), which integrates clinical data with patient-reported symptoms, classifying nutritional status (A–well-nourished, B–moderately malnourished, C–severely malnourished) and assigning a risk score (0–50). Azzeh et al. [50] applied the Modified Subjective Global Assessment (M-SGA), combining clinical history and physical examination, with scores ranging from 7 (well-nourished) to 35 (severely malnourished), demonstrating its reliability for early detection and nutritional monitoring in hemodialysis populations.

Tang and Fu [52] used the standard Subjective Global Assessment (SGA), a validated method widely applied in chronic disease contexts, classifying patients similarly to PG-SGA (A, B, C categories). Wang et al. [51] employed three tools: the Nutritional Risk Screening 2000 (NRS2000), which evaluates nutritional status, disease severity, and age (score ≥3 indicating risk); the Malnutrition Inflammation Score (MIS); and the Modified Quantitative SGA (MQSGA), offering a multidimensional approach to identify malnutrition risk and guide targeted nutritional interventions.

The Malnutrition Inflammation Score (MIS) evaluates malnutrition associated with chronic inflammation through four domains: 1) clinical history (weight loss, intake, GI symptoms, functional status); 2) physical exam (edema, subcutaneous fat, muscle mass); 3) BMI; and 4) laboratory parameters (serum albumin, CRP, transferrin). Higher MIS scores indicate greater nutritional risk. The Modified Quantitative Subjective Global Assessment (MQSGA) is a structured, numerical adaptation of the SGA, integrating clinical history and physical exam, with scores ranging from 7 (well-nourished) to ≥14 (severely malnourished).

Seven studies did not employ direct nutritional assessment tools, focusing instead on related dimensions, such as quality of life [46], health literacy [47], self-care, and self-efficacy [49,53]. Treatment adherence by Arad et al. [48], evaluating adherence across four domains: hemodialysis attendance, medication, fluid, and diet, complemented by laboratory markers.

## 4 Discussion

According to the data presented, there is a high percentage of malnutrition in the hemodialysis patient population, with 54.5 % of participants showing signs of malnutrition (51.7 % moderately and 2.8 % severely malnourished) [50]. Malnutrition is strongly associated with higher morbidity and mortality rates and a significant reduction in quality of life [56].

Protein-energy wasting and micronutrient deficiencies, including vitamins (e.g., vitamin D) and minerals (e.g., iron, calcium, sodium), contribute to muscle weakness, bone disorders, and cardiovascular problems in hemodialysis patients [57]. Chronic inflammation exacerbates these deficits by increasing catabolism and reducing appetite, while uremia and dialysis-related complications can lead to nausea, vomiting, taste changes, and reduced calorie intake [58]. Low adherence to dietary recommendations has also been associated with dyslipidemia, higher uric acid and C-reactive protein levels, and insulin resistance [59]. Another study argues that hemodialysis patients have difficulties completing activities of daily living and adhering to food and fluid restrictions due to decreased self-care capacity and loss of skills [60].

Studies such as those by Hosseini et al. [53], Chiang et al. [54], Wang et al. [55], and Pack & Lee [49] have highlighted the potential of technology-based interventions using mobile applications and digital platforms that have improved biochemical indicators, increased self-efficacy, and quality of life. Hosseini et al. [53] described statistically significant improvements in self-efficacy and self-care performance after implementing an educational app. Similarly, Pack & Lee [49] demonstrated that a program based on a digital application significantly reduced phosphorus and potassium levels, critical indicators of the nutritional status of HD patients, compared to conventional interventions. Chiang et al. [54] also showed that using a digital application for individualized phosphorus control increased knowledge about diet and improved biochemical indicators. In contrast, Wang et al. [55] showed that ICBT increased knowledge, attitude, and practice scores. These interventions enable real-time feedback, personalization of education, and continuous monitoring, consistent with other evidence pointing to the benefits of digital technologies in chronic disease management. For their part, Arad et al. [48] illustrate the combination of face-to-face education with telephone and SMS follow-up, boosting results. It reconciles the benefits of direct contact with the advantages of remote monitoring, emphasizing that including technological elements (such as telephone calls and SMS) increases the effectiveness of interventions through telenursing. Non-technological interventions such as those applied by Tang & Fu [52] and Mozafari et al. [47] show that face-to-face educational strategies (e.g., teach-back and the use of pictorial images) also promote improvements in nutritional knowledge and adherence to restrictions. However, they may depend more on interpersonal contact and direct communication (Appendix D).

In addition, the observational study by Azzeh et al. [50] makes an important contribution by identifying essential predictors, such as unemployment, low muscle strength, high drug consumption, and longer time on dialysis, which allow for preventive and personalized interventions.

Based on the comparative analysis of the included studies, marked differences are observed between middle-aged and older adults regarding educational and behavioral interventions aimed at promoting self-care and nutritional control. Middle-aged adults demonstrated high adaptability to mobile technologies, such as smartphone apps, which significantly contributed to improvements in treatment adherence, quality of life, and biochemical indicators (e.g., potassium, phosphorus, and albumin). Behavioral models such as the Transtheoretical Model and Cognitive Behavioral Therapy (CBT) effectively enhanced this age group's knowledge, self-efficacy, and psychosocial outcomes. In contrast, older adults showed better results with structured and visual educational approaches, such as pictorial methods and teach-back strategies, which align with their cognitive preferences and technological limitations. Although physiological responses in older adults tend to be slower, biochemical benefits were observed when interventions were personalized and cognitively accessible. The study by Chiang et al. [54] reinforces the importance of personalization, showing that even among older participants, technological use can be successful when adapted to individual needs and supported appropriately. These findings underscore the relevance of age-appropriate health interventions—both in format and complexity—to optimize clinical and behavioral outcomes [61].

Most successful interventions, such as those by Mackay et al. [51] and Wang & Hu [55], involved multidisciplinary teams, including nurses, nutritionists, nephrologists, and even psychologists. This collaborative approach ensures comprehensive content and support. Effectiveness was evaluated using a combination of objective and subjective measures. This included changes in laboratory parameters (e.g., potassium, phosphorus, creatinine, albumin), self-reported questionnaires (quality of life, self-efficacy, knowledge), and adherence rates. Regular assessments (e.g., monthly, at 2-month, 6-month, or 12-month follow-ups) were crucial for monitoring short-term and sustained effects. However, many of the studies included in this review demonstrate the effectiveness of technological nursing interventions over relatively short periods (typically up to 3–6 months) [46,47,53,55]. However, CKD is a progressive, long-term condition where the sustainability of behavioral changes and the lasting impact on clinical outcomes (such as reduced hospitalizations, improved long-term quality of life, and decreased morbidity and mortality) are critically important. The absence of long-term follow-up data limits our understanding of the durability of these interventions’ benefits.

## 5 Limitations and strengths

The small number of published studies and the small sample sizes limit the generalizability of the results and the ability to identify clinically significant differences. The heterogeneity of the study designs makes it difficult to make direct comparisons and synthesize uniform conclusions (biochemical indicators, self-efficacy, literacy). The duration of the interventions, with a lack of long-term evaluation, and many studies presenting short- or medium-term follow-ups, prevents assessment of the sustainability of the long-term effects of the interventions, a crucial aspect considering the chronic nature of kidney disease.

As for the limitations of the evidence included in this review, the selection of studies may have been influenced by language restrictions and the availability of complete publications in selected databases, leading to the exclusion of relevant evidence and contributions. The research was conducted mainly in Asia, Australia, and Iran. This regional concentration limits the generalizability of the findings to other cultural, economic, and healthcare contexts worldwide.

This research demonstrates several strengths, including a comprehensive and age-stratified evidence synthesis across diverse populations. By distinguishing between middle-aged and older adults, the study captures age-specific nuances in technology use, cognitive engagement, and physiological responses to intervention dimensions often overlooked in more generalized analyses. Furthermore, integrating objective outcomes (e.g., biochemical markers) and subjective measures (e.g., self-efficacy, knowledge, and psychosocial responses) allows for a multidimensional understanding of intervention effectiveness. Finally, the inclusion of recent studies ensures the contemporary relevance of the findings and highlights the evolving role of digital health tools in the management of chronic conditions.

## 6 Conclusion

The systematic review identified that the most effective nursing interventions for promoting nutritional self-care in patients with hemodialysis involve integrating technology and innovation. The use of mobile applications, communication methodologies, education, and continuous monitoring by the nursing team enhances patient engagement in managing chronic kidney disease and hemodialysis treatment, improving self-care and quality of life.

Combining digital approaches (mobile apps, e-learning, ICBT) with face-to-face methods has shown promising results, highlighting the need for appropriate training of nurses to integrate technology into their practice effectively. For future research, focusing on long-term results is suggested, as they are essential for determining the durability of the benefits obtained and the evolution of self-care in this population. It is also recommended that new technologies be explored, integrating artificial intelligence and real-time feedback systems, to develop malnutrition prevention and nutritional risk stratification programs.

## Data availability statement

The datasets generated during and/or analyzed during the current study are available from the corresponding author upon reasonable request.

## CRediT authorship contribution statement

**Amélia Pernas:** Conceptualization, Methodology, Validation, Formal analysis, Investigation, Data curation, Writing – original draft, Writing – review & editing. **Sara Pires:** Conceptualization, Methodology, Writing – review & editing, Methodology, Validation, Formal analysis. **Idalina Gomes:** Conceptualization, Methodology, Validation, Writing – review & editing, Supervision**,** Funding acquisition. **César Fonseca:** Conceptualization, Methodology, Validation, Writing – review & editing, Supervision. **Ana Ramos:** Conceptualization, Methodology, Validation, Investigation, Data curation, Formal analysis, Supervision, Writing - original draft, Writing – review & editing, Project administration.

## Funding

This work is funded by national funds through the Foundation for Science and Technology, under the project UIDB/04923.

## Declaration of competing interest

The authors declare there is no conflict of interest.
