# Supplementary material for: Technological nursing interventions on nutritional status of middle-aged and older adults undergoing hemodialysis: A systematic review
Source: Int J Nurs Sci. 2025 Aug 14;12(5):493–500. doi: 10.1016/j.ijnss.2025.08.008 (PMC12504971; doi:10.1016/j.ijnss.2025.08.008)
Supplement: Multimedia component 1 [file mmc1.docx]

信息化护理干预对血液透析患者营养状况的影响：系统综述

Amélia Pernas，Sara Pires, Idalina Gomes, César Fonseca, Ana Ramos

【摘要】

**目的** 营养不良在血液透析患者中较为常见，会增加死亡率，且显著影响其生活质量。该研究描述能够促进自我护理并改善中老年血液透析患者营养状况的信息化护理干预措施。

**方法**  对Medline、CINAHL、the Cochrane Library、Scopus和Web of Science数据库进行检索。检索时间为2018年至2024年，纳入经过同行的以葡萄牙语、英语或西班牙语发表的文献及灰色文献，研究对象为40岁及以上接受定期血液透析的患者，采用澳大利亚Joanna Briggs Institute循证卫生保健中心文献质量评价工具对纳入研究的方法学质量进行评价。

**结果** 共检出738篇文献，10篇符合纳入标准。总结信息化护理干预措施的5个方面：1) 具备营养数据库、食物记录和个性化反馈等功能的移动应用程序和数字平台；2) 通过社交媒体和聊天工具进行的在线学习和虚拟教育；3) 采用面对面、电话和短信沟通的混合随访模式；4) 利用多学科团队专注于营养状况的宣教策略，采用回授法和图示教学法等； 5) 患者治疗依从性和实验室指标的综合评估工具。8项研究显示患者生化指标（如磷、钠、钾、钙、铁、白蛋白、尿素和血红蛋白）及营养状况有所改善，3项研究显示患者自我效能感增强，2项研究显示患者生活质量改善。

**结论** 信息化护理干预措施的实施有助于改善血液透析患者营养状况，这凸显了制订综合策略以提高患者治疗依从性并预防营养不良的重要性。

【关键词】血液透析；营养状况；护理；患者；自我护理；技术创新

通信作者：Ana Ramos, E-mail: [anaramos@esel.pt](mailto:anaramos@esel.pt)
