# Supplementary material for: Technological nursing interventions on nutritional status of middle-aged and older adults undergoing hemodialysis: A systematic review
Source: Int J Nurs Sci. 2025 Aug 14;12(5):493–500. doi: 10.1016/j.ijnss.2025.08.008 (PMC12504971; doi:10.1016/j.ijnss.2025.08.008)
Supplement: Multimedia component 2 [file mmc2.docx]

Appendix A Search strategy

| Database | Search statement | Results | |
| --- | --- | --- | --- |
|  |  | Without limiters | With Limiters: year of publication |
| CINAHL  Ultimate (EBSCOhost) | ((Renal Insufficiency Chronic) OR (Kidney Failure, Chronic) OR hemodialysis OR (dialysis patient)) AND ((Nurs* OR (Digital Technology) OR telenursing OR (Artificial Intelligence) OR (Mobile health) OR Telemedicine OR technology) OR (Self- management OR (self care) OR Self- efficacy)) AND ((nutritional status) OR (nutritional assessment)) | Total = 129 | Total = 45 |
| MEDLINE  (EBSCOhost) | ((Renal Insufficiency Chronic) OR (Kidney Failure, Chronic) OR hemodialysis OR (dialysis patient)) AND ((Nurs* OR (Digital Technology) OR telenursing OR (Artificial Intelligence) OR (Mobile health) OR Telemedicine OR technology) OR (Self- management OR (self care) OR Self- efficacy)) AND ((nutritional status) OR (nutritional assessment)) | Total = 300 | Total = 162 |
| Cochrane Central Register of Controlled Trials  (EBSCOhost) | ((Renal Insufficiency Chronic) OR (Kidney Failure, Chronic) OR hemodialysis OR (dialysis patient)) AND ((Nurs* OR (Digital Technology) OR telenursing OR (Artificial Intelligence) OR (Mobile health) OR Telemedicine OR technology) OR (Self- management OR (self care) OR Self- efficacy)) AND ((nutritional status) OR  (nutritional assessment)) | Total = 55 | Total = 33 |
| Scopus | (ALL(renal AND insufficiency AND chronic OR kidney AND failure, AND chronic OR hemodialysis OR dialysis AND patient) AND ALL(nurs* OR digital AND technology OR telenursing OR artificial AND intelligence OR mobile AND health OR telemedicine OR technology OR self- management OR self AND care OR self- efficacy) AND ALL(nutritional AND status OR nutritional AND assessment)) | Total = 220 | Total = 120 |

| Web of Science | (((((((((((((((((ALL = (Renal Insufficiency Chronic)) OR ALL = (Kidney Failure, Chronic)) OR ALL = (hemodialysis)) OR ALL = (dialysis patient)) AND ALL = (Nurs* )) OR ALL = (Digital Technology)) OR ALL = (telenursing)) OR ALL = (Artificial Intelligence)) OR ALL = (Mobile health)) OR ALL = (Telemedicine)) OR ALL = (technology)) OR ALL = (Self-management )) OR ALL = (self care)) AND ALL = (nutritional status)) OR ALL = (nutritional assessment)) AND ALL = (Nutritional status)) AND ALL = (nursing care) | Total = 34 | Total = 18 |
| --- | --- | --- | --- |
| Google Scholar | (“Hemodialysis”) AND (“Nursing Care”) AND (“Self-care”) AND (“Nutritional Status”) AND (“Technology”) | Total = 243 | Total = 113 |

Appendix B JBI Critical Appraisal

| JBI Critical Appraisal Tool for randomized controlled trials | | | | | | | | | | | | | | | | |
| --- | --- | --- | --- | --- | --- | --- | --- | --- | --- | --- | --- | --- | --- | --- | --- | --- |
| Study | Rev | Items | | | | | | | | | | | | | Score（%） | Level |
|  |  | 1 | 2 | 3 | 4 | 5 | 6 | 7 | 8 | 9 | 10 | 11 | 12 | 13 |  |  |
| Naseri-Salahshour et al., (2020) | AP | Y | Y | Y | Y | N | Y | Y | Y | Y | Y | Y | Y | Y | 92.3 |  |
|  | AR | Y | Y | Y | Y | N | Y | Y | Y | Y | Y | Y | Y | Y |  | High |
| Mozafari et al., (2024) | AP | Y | Y | Y | Y | N | N | Y | Y | Y | Y | Y | Y | Y | 84.6 | High |
|  | AR | Y | Y | Y | Y | N | N | Y | Y | Y | Y | Y | Y | Y |  |  |
| Arad et al., (2021) | AP | Y | Y | Y | Y | N | Y | Y | Y | Y | Y | Y | Y | Y | 92.3 | High |
|  | AR | Y | Y | Y | Y | N | Y | Y | Y | Y | Y | Y | Y | Y |  |  |
| Pack & Lee (2021) | AP | Y | N | Y | N | N | Y | Y | Y | Y | Y | Y | Y | Y | 76.9 | Medium |
|  | AR | Y | N | Y | N | N | Y | Y | Y | Y | Y | Y | Y | Y |  |  |

*Note*：Y = Yes; N = No; U = Unclear; NA = Not Applicable. Items from JBI Critical appraisal tool for Randomized Controlled Trials as follow. 1. Was true randomization used for assignment of participants to treatment groups? 2.Was allocation to treatment groups concealed? 3.Were treatment groups similar at the baseline? 4. Were participants blind to treatment assignment? 5.Were those delivering treatment blind to treatment assignment? 6.Were outcomes assessors blind to treatment assignment? 7.Were treatment groups treated identically other than the intervention of interest? 8.Was follow-up complete and if not, were differences between groups in terms of their follow-up adequately described and analyzed? 9.Were participants analyzed in the groups to which they were randomized? 10. Were outcomes measured in the same way for treatment groups? 11.Were outcomes measured in a reliable way? 12.Was appropriate statistical analysis used? 13.Was the trial design appropriate, and any deviations from the standard RCT design (individual randomization, parallel groups) accounted for in the conduct and analysis of the trial?

| JBI Critical appraisal tool of analytical cross-sectional studies | | | | | | | | | | | |
| --- | --- | --- | --- | --- | --- | --- | --- | --- | --- | --- | --- |
| Study | Rev | Items | | | | | | | | Score (%) | Level |
|  |  | 1 | 2 | 3 | 4 | 5 | 6 | 7 | 8 |  |  |
| Azzeh et al., (2022) | AP | Y | Y | Y | Y | Y | N | Y | Y | 87.5 | High |
|  | AR | Y | Y | Y | Y | Y | N | Y | Y |  |  |

*Note*：Y = Yes; N = No; U = Unclear; NA = Not Applicable. Items from Critical appraisal of analytical cross sectional studies as follow. 1= Were the criteria for inclusion in the sample clearly defined? 2 = Were the study subjects and the setting described in detail? 3 = Was the exposure measured in a valid and reliable way? 4 = Were objective, standard criteria used for measurement of the condition? 5 = Were confounding factors identified? 6 = Were strategies to deal with confounding factors stated? 7 = Were the outcomes measured in a valid and reliable way? 8 = Was appropriate statistical analysis used?

| JBI Critical appraisal tool of Quasi-experimental studies | | | | | | | | | | | | |
| --- | --- | --- | --- | --- | --- | --- | --- | --- | --- | --- | --- | --- |
| Study | Rev | Items | | | | | | | | | Score (%) | Level |
|  |  | 1 | 2 | 3 | 4 | 5 | 6 | 7 | 8 | 9 |  |  |
| Mackay et al., (2019) | AP | Y | NA | N | Y | Y | Y | Y | N | Y | 75.0 | Medium |
|  | AR | Y | NA | N | Y | Y | Y | Y | N | Y |  |  |
| Tang & Fu (2021) | AP | Y | Y | Y | Y | Y | Y | Y | N | Y | 88.9 | High |
|  | AR | Y | Y | Y | Y | Y | Y | Y | N | Y |  |  |
| Hosseini et al., (2023) | AP | Y | N | NA | NA | Y | Y | Y | NA | Y | 83.3 | High |
|  | AR | Y | N | NA | NA | Y | Y | Y | NA | Y |  |  |
| Chiang et al., (2021) | AP | Y | Y | N | Y | Y | Y | Y | Y | Y | 88.8 | High |
|  | AR | Y | Y | N | Y | Y | Y | Y | Y | Y |  |  |
| Wang, & Hu (2024) | AP | Y | Y | N | Y | Y | Y | Y | Y | Y | 88.9 | High |
|  | AR | Y | Y | N | Y | Y | Y | Y | Y | Y |  |  |

*Note*：Y=Yes; N=No; U=Unclear; NA=Not Applicable. Items from JBI Critical appraisal tool for Quasi-experimental studies as follow. 1. Is it clear in the study what is the “cause” and what is the “effect” (i.e. there is no confusion about which variable comes first)? 2. Was there a control group? 3. Were participants included in any comparisons similar? 4. Were the participants included in any comparisons receiving similar treatment/care, other than the exposure or intervention of interest? 5. Were there multiple measurements of the outcome, both pre and post the intervention/exposure? 6. Were the outcomes of participants included in any comparisons measured in the same way? 7. Were outcomes measured in a reliable way? 8. Was follow-up complete and if not, were differences between groups in terms of their follow-up adequately described and analyzed? 9. Was appropriate statistical analysis used?

Appendix C Characteristics of the included studies

| Study and location | Aim of this study | Study Design | Sample Size  Age  Group activities | Measures and instruments | Intervention | Key findings |
| --- | --- | --- | --- | --- | --- | --- |
| Mackay, et al. [51] (2019)  Australia | To establish a nutrition service in a hemodialysis (HD) unit using evidence and implementation science | Observational study | 33 patients on HD (16 women); Mean age: 63.7 years  Group activities: Monthly multidisciplinary reviews and ongoing education for staff and patients; dietitian support included mentoring and regular evaluation over 18 months. | Scored Patient-Generated Subjective Global Assessment (PG-SGA) | Implementation of Knowledge-to-Action framework; 10 in-person workshops for 148 nurses; professional development for nutritionists; monthly team meetings and assessments. | Improved nutritional status over time; well-nourished increased from 71.9 % to 80 %; malnutrition reduced from 28 % to 20% over 12 months: 0 % |
| Azzeh, et al. [50] (2022)  Arabia Saudita | To assess the nutritional status of HD patients and identify associated factors. | Cross-sectional study | 211 adults on HD (42.2 % women), with a mean age of 46.4 years.  Group activities: Not applicable | Modified-subjective Global Assessment (M-SGA)  Biochemical: Hb, albumin  Bioimpedance: BMI, fat-free mass.  GRIPX Digital Hand Dynamometer Grip Strength Measurement | The assessment focused on nutritional predictors and muscle condition. | Malnutrition is associated with lower handgrip strength and lower lean mass. More than 4 medications intake significantly increased malnutrition risk. More than 4 years on HD increased malnutrition risk by 11.36 times. Unemployment increased risk between 1.18–4.30. |
| Naseri-Salahshour, al. [46] (2020)  Iran | To determine the effect of healthy nutrition through virtual social networks on the quality of life of individuals undergoing HD | Randomized controlled trials | 94 HD patients (intervention group: *n =* 48; 59.64 years, *SD =* 21.27; 21.29 % female. Control group*: n =* 46; 57.25 years, *SD =* 20.21, 18.08 % female).  Group Intervention: Telegram-based nutrition education over 4 weeks; 2 messages/week with daily feedback provided by researchers. | Kidney Disease Quality of Life Instrument (KDQOL)  Sodium, potassium, phosphorus, calcium, and magnesium levels | Virtual education program focused on dietary management for HD (fluid restriction, electrolytes, food groups). | Significant improvements were observed in quality of life, along with reductions in serum sodium, potassium, phosphorus, and magnesium levels. No significant change was found in serum calcium levels. The intervention proved effective in the short term. |
| Mozafari et al. [47] (2024)  Iran | To compare the effects of two educational strategies, teach-back versus pictorial image-based education, on the understanding of dietary restrictions in HD patients. | Randomized controlled trials | 69 participants; mean age: 68.8 years, with 36 women.  Group activities: diet education comprising four 20–30 min sessions, with assessment 2 months after the intervention. | Pre- and post-intervention knowledge questionnaire.  Laboratory data (potassium, phosphorus, creatinine, blood urea nitrogen) extracted from clinical records. | Educational sessions on nutrition in HD are conducted by nurses, nutritionists, and nephrologists, divided into three groups: a pictorial image-based education group, a teach-back education group, and a control group (receiving standard care). | The Teach-Back group showed reductions in potassium and phosphorus levels. The pictorial group showed improvements in creatinine and blood urea nitrogen. Structured educational interventions were effective in improving dietary knowledge and clinical outcomes among HD patients. |
| Tang & Fu [52] (2021)  China | To investigate the effects of combining the Transtheoretical Model with a nutritional intervention in patients undergoing HD. | Observational study | 100 participants. Group control: 58.96 *±* 3.19 years, with 26.5 % females. Group intervention: 59.02 ± 3.17 years, with 25.5 females.  Group activities: Monthly seminars reinforced patient behaviors. Nurses regularly engaged caregivers to enhance patient support. | Renal Adherence Attitudes Questionnaire (RAAQ)  Renal Adherence Behavior Questionnaire (RABQ)  Subjective Global Assessment (SGA)  Anthropometric indicators: creatinine, blood urea nitrogen (BUN), mid-arm muscle circumference (MAMC), triceps skinfold (TSF), body mass index (BMI), urea clearance index (KT/V), and urea reduction ratios (URR%) | The intervention followed the stages of the Transtheoretical Model, encompassing contemplation, preparation, action, and maintenance, with a focus on promoting behavioral change related to dietary habits in HD patients. | Patients in the group that received the combined intervention showed improved nutritional status and better adherence to dietary recommendations. It enhanced clinical outcomes, including improved laboratory values and dialysis adequacy. |
| Hosseini et al., [53] (2023)  Iran | To determine the impact of education through an app on self-care and self-efficacy in hemodialysis patients. | Quasi-experimental | 60 hemodialysis patients, with a mean age of 39.26 ± 9.24 years, and 44.4 % were women.  Group activities: over six months, patients could use the app at different times, with a monthly re-evaluation. | Self-care and self-efficacy questionnaires.  Analysis of different domains of self-efficacy (e.g., stress reduction, positive attitude). | The mobile app Diapass contained educational content based on Orem’s Self-Care Model and covered topics including nutrition, infection prevention, medication, complications, physical activity, sleep, rest, and sexual health. | Participants showed improved self-care and self-efficacy after using the mobile app. The most notable improvements were observed in areas such as stress management and the development of a positive attitude. |
| Chiang et al., [54] (2021)  China | To evaluate the effectiveness of a personalized dietary phosphate control program using a smartphone application in the treatment of hyperphosphatemia. | Quasi-experimental study | 60 HD patients, with an age range of 30 to 70 years.  Group activities: Mobile app recorded and summarized scores regularly, offered personalized recipes and cooking tips, and allowed the care team to track food records and diet adjustments. | Phosphate-related dietary knowledge test  Self-efficacy questionnaires  Serum Phosphate and albumin levels | Personalized education via a mobile app, which included features such as a nutritional database, food logging, phosphate-to-protein ratio scoring, educational recipes, and customized dietary feedback. The control group followed conventional education based on printed materials and verbal counseling. | Participants with the app-assisted demonstrated better outcomes in phosphate control, greater understanding of dietary phosphorus management, and higher self-efficacy. Older and married participants showed higher levels of phosphate knowledge after the intervention. |
| Wang, & Hu [55]  (2024)  China | To evaluate the impact of Internet-based Cognitive Behavioral Therapy (ICBT) on the nutritional status and quality of life of HD patients. | Randomized controlled trials | 220 patients in an HD program (110 in each group). Group experimental: average of 45.6 years, with 54 females. Group control: 43.9 years, with 58 females)  Sex:  Group activities: 3 months WeChat mini program, with daily available counseling. | Nutritional Knowledge-attitudes and practices (KAP) for HD patients  Biochemical indicators  Modified Quantitative Subjective Global Assessment (MQSGA)  Nutritional Risk Screening 2000 (NRS2000)  MIS (Malnutrition-Inflammation Score) | The ICBT group used a WeChat-based application and received educational content (videos, presentations); cognitive restructuring sessions, behavioral and dietary tracking; relaxation training (music therapy and progressive muscle relaxation); psychological support; nutritional monitoring and lab result discussions. | Internet-based cognitive behavioral therapy improved nutritional status, such as higher blood calcium, albumin, and prealbumin levels, increased arm circumference, and lower potassium and phosphorus levels. Reduce nutritional risk and inflammation scores, enhance appetite, and improve biochemical markers, knowledge, attitude, and practice assessments. |
| Arad et al. [48] (2021)  Iran | To evaluate the effects of a patient education program for HD patients and nurse-led telephone follow-up on treatment adherence. | Randomized controlled trials | 66 HD patients, divided into a control group (*n =* 33; 30 years ± 9,5; 42.4 % female) and an intervention group *(n =* 33; 27 ± 11.5 years; 45.5 female)  Group activities: 36 sessions/20 min/12 weeks; Phone calls and one educational text message per day on topics, totaling 90 messages over the intervention period | Biochemical indicators  End-Stage Renal Disease Adherence Questionnaire (ESRD-AQ) | A technology-assisted nursing intervention using telephone counseling and text messaging for over three months to support patient education (diet, medication, and fluid restrictions) and adherence. | The intervention group showed significantly improved adherence to HD, medication, fluid, and diet regimen at all time points. Lab results (calcium and iron) also improved post-intervention, confirming the effectiveness of the SMS-based. |
| Pack & Lee [49] (2021)  South Korea | To develop a dietary self-management program based on a smartphone application for HD patients and examine its effects on biochemical parameters, indicators, self-efficacy, and quality of life. | Randomized controlled trials | 75 patients (37 in the experimental group and 38 in the control group), with a total mean of 51.3 ± 9.6 years; 38.7 % women. Group activities: Initial training, guided practice using the app with real-time feedback, and ongoing support. Eight weeks with face-to-face training (30 minutes per day for 3 days a week) and online counseling | Biochemical indicators: serum phosphorus, potassium, and albumin.  Self-efficacy questionnaire for hemodialysis patients.  KDQOL-SF | Three phases: initial training on dietary self-management and app use, followed by app-based practice with professional support and feedback, and a maintenance phase that included continued self-management and periodic laboratory monitoring. | The program delivered through the smartphone app led to greater improvements in serum phosphorus and potassium levels, self-efficacy, and quality of life over time. However, changes in albumin levels were similar between both groups. |

8


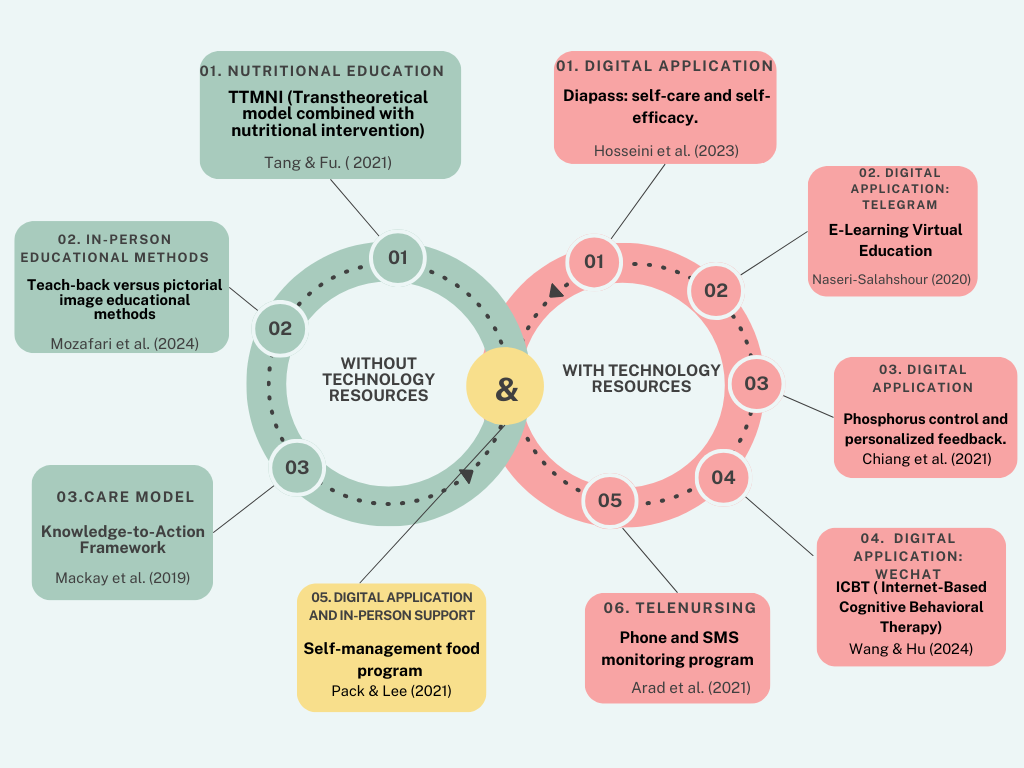


Appendix D. Synthesis of the nursing intervention
